# Cerebral Apolipoprotein-D Is Hypoglycosylated Compared to Peripheral Tissues and Is Variably Expressed in Mouse and Human Brain Regions

**DOI:** 10.1371/journal.pone.0148238

**Published:** 2016-02-01

**Authors:** Hongyun Li, Kalani Ruberu, Tim Karl, Brett Garner

**Affiliations:** 1 Illawarra Health and Medical Research Institute, University of Wollongong, Wollongong, NSW 2522, Australia; 2 School of Biological Sciences, University of Wollongong, Wollongong, NSW 2522, Australia; 3 Neuroscience Research Australia, Randwick, NSW 2031, Australia; 4 School of Medical Sciences, University of New South Wales, Sydney, NSW 2052, Australia; 5 Schizophrenia Research Institute, Randwick, NSW 2031, Australia; Consejo Superior de Investigaciones Cientificas, SPAIN

## Abstract

Recent studies have shown that cerebral apoD levels increase with age and in Alzheimer’s disease (AD). In addition, loss of cerebral apoD in the mouse increases sensitivity to lipid peroxidation and accelerates AD pathology. Very little data are available, however, regarding the expression of apoD protein levels in different brain regions. This is important as both brain lipid peroxidation and neurodegeneration occur in a region-specific manner. Here we addressed this using western blotting of seven different regions (olfactory bulb, hippocampus, frontal cortex, striatum, cerebellum, thalamus and brain stem) of the mouse brain. Our data indicate that compared to most brain regions, the hippocampus is deficient in apoD. In comparison to other major organs and tissues (liver, spleen, kidney, adrenal gland, heart and skeletal muscle), brain apoD was approximately 10-fold higher (corrected for total protein levels). Our analysis also revealed that brain apoD was present at a lower apparent molecular weight than tissue and plasma apoD. Utilising peptide N-glycosidase-F and neuraminidase to remove N-glycans and sialic acids, respectively, we found that N-glycan composition (but not sialylation alone) were responsible for this reduction in molecular weight. We extended the studies to an analysis of human brain regions (hippocampus, frontal cortex, temporal cortex and cerebellum) where we found that the hippocampus had the lowest levels of apoD. We also confirmed that human brain apoD was present at a lower molecular weight than in plasma. In conclusion, we demonstrate apoD protein levels are variable across different brain regions, that apoD levels are much higher in the brain compared to other tissues and organs, and that cerebral apoD has a lower molecular weight than peripheral apoD; a phenomenon that is due to the N-glycan content of the protein.

## Introduction

Apolipoprotein D (apoD) is an ~ 29 kDa glycoprotein member of the lipocalin family [1]. ApoD is well known as a plasma protein that associates with the high-density lipoprotein (HDL) fraction [2, 3]. The apoD crystal structure reveals an eight-stranded antiparallel β-barrel flanked by an α-helix [4]. Similar to other lipocalins, the β-barrel encloses a conically shaped internal hydrophobic cavity that functions as a ligand-binding site for small hydrophobic molecules. Early studies suggested that apoD binds a range of lipids including arachidonic acid (AA), cholesterol and several steroids [5–8]. More recent studies indicate that binding of lipids in the apoD binding pocket is quite specific [4, 9]. Progesterone, AA and retinoic acid bind to apoD with high affinity whereas pregnenolone and specific eicosanoids (e.g. 12-HETE and 5,15-diHETE) bind with reduced affinity [6–8, 10].

In addition to its expression in plasma, apoD is highly expressed in the central and peripheral nervous systems [11, 12]. Recent studies indicate a role for apoD in protection against lipid peroxidation in the brain. Loss-of-function Drosophila mutants for the apoD homolog glial lazarillo (GLaz) were found to be more sensitive to oxidative stress and contained higher concentrations of lipid peroxidation products [13]. In addition, over-expression of GLaz in transgenic Drosophila lines increased resistance to oxidative stress, extended lifespan and protected against hyperoxia-induced behavioural decline [14]. Expression of human apoD in old flies also reduced the accumulation of aldehydic end-products of lipid peroxidation [15]. We have identified an antioxidant mechanism of action for apoD that is based on the reduction of reactive lipid hydroperoxides to relatively inert lipid hydroxides; a reaction that requires conversion of apoD methionine residue 93 to methionine sulfoxide [16, 17].

Studies in mice have confirmed that brain apoD is induced in response to oxidative stress, that lipid peroxidation is increased in the brains of apoD null mice, and that expression of human apoD prevents lipid peroxidation in response to paraquat-induced oxidative stress in the brain [18]. More recent studies have sown that loss of apoD in the APP/PS1 amyloidogenic Alzheimer’s disease (AD) mouse model significantly worsens AD pathology, whereas neuronal expression of human apoD in the same AD mouse model reduces AD pathology [19]. These observations, along with the known induction of apoD in affected brain regions in human AD and mouse models [20–26], underscore the importance of defining possible difference in apoD expression in different brain regions that may be susceptible to oxidative stress and neurodegenerative conditions.

Previous studies have quantitatively assessed the expression of apoD mRNA levels in the brain and other tissues, and there are several reports demonstrating the presence of apoD protein in specific brain areas such as the hippocampus, cerebellum, dorsolateral pre-frontal cortex and substantia nigra. Pioneering studies by Navarro *et al*. [27], Terrisse *et al*. [20] and Thomas *et al*. [22, 28] provided initial evidence that apoD protein levels may vary in specific cell types and across brain regions in human post-mortem brain tissues. Navarro *et al*. used immunostaining methods to demonstrate apoD immunoreactivity in neurons and glia of the cerebellar cortex and showed that neurons do not express apoD in a uniform staining pattern [27]. In addition they reported that in the frontal, parietal and temporal cortices, neuronal apoD was not observed [27]. Terrisse *et al*. demonstrated the presence of apoD in the human hippocampus by western blotting and found that levels were significantly increased in AD postmortem samples compared to control samples [20]. Subsequent publications by Thomas *et al*. quantified brain apoD protein levels by an ELISA method [22, 28]. Initially this group compared apoD levels in six brain regions from healthy controls and Schizophrenia patients and discovered an approximate doubling of apoD levels in the dorsolateral prefrontal cortex of the Schizophrenia patients [28]. Their follow-up study used the same ELISA method to show that apoD levels were increased in the prefrontal cortex of AD patients [22].

Although several studies have assessed the expression of apoD mRNA and protein levels in various brain regions [11, 12, 19, 20, 25, 27, 29–34] there are limited data available regarding the quantitative expression of apoD protein levels across different brain regions, how this may relate in mice to other organs, and how differences in apoD molecular weight (that may be present in cerebral versus peripheral sources in mice and humans) might relate to alterations in the extent of N-linked glycosylation. In the current study, we have addressed these questions by undertaking western blot analysis of apoD in various brain regions and organs and by utilising glycosidase digestion techniques to investigate apoD molecular weight heterogeneity.

## Materials and Methods

### Animals

All mice were on a pure (backcrossed > 10 generations) C57BL6 background. Test animals were 2 males and 1 female at ~8 months of age (n = 3, 239 ± 11 days old, mean ± SE). Animal ethics approval was from the University of Wollongong Animal Ethics Committee (AE11/03).

### Human brain tissue

The human tissues were derived from a cohort of aged healthy control donor brains that have been previously described in detail [26]. The samples analysed in the present study were comprised of 1 male and 2 female donors (79 ± 6 years old, post-mortem interval 24 ± 11 h, means ± SE).

### Ethics statement

Human brain tissue was supplied by the New South Wales Tissue Resource Centre at the University of Sydney and the Sydney Brain Bank (http://www.nswbrainbanknetwork.org.au/). A plasma sample was obtained from a healthy 50 year old male volunteer. Written informed consent from the donor or the next of kin was obtained for the use of the samples in research. The use of human brain tissue was approved by the University of Wollongong Human Research Ethics Committee (HE10/327). The research was conducted according to the principles expressed in the Declaration of Helsinki. The three human control brain tissue samples used in the present study were part of a larger cohort of samples previously described in detail [26]. The use of mice in this study was approved by the University of Wollongong Animal Ethics Committee (AE11/03). Mice were euthanized by CO_2_ asphyxiation.

### Sample collection and processing

Mice were euthanized by CO_2_ asphyxiation and transcardially perfused with ice-cold phosphate buffered saline (PBS). The brains were removed and sagittally divided and the olfactory bulb, hippocampus, frontal cortex, striatum, cerebellum, brain stem and thalamus/hypothalamus (the thalamus/hypothalamus regions were combined) were dissected from the right hemisphere before snap freezing and storage at -80°C. Brain tissues were processed by accurately weighing ~15 mg of frozen tissue that was homogenized in 12 volumes of 140 mM NaCl, 3 mM KCl, 25 mM Tris (pH 7.4), containing 1% Nonidet P-40, 100μM PMSF and Sigma protease inhibitor cocktail (TBS/NP40 extraction buffer) using a Precellys 24 homogenizer (2 x 30s, 6000 *g*). Homogenates were centrifuged at 16,000 *g* for 30 min at 4°C and the TBS/NP40-soluble supernatant collected and stored at −80°C until use in western blotting experiments. Protein concentration was measured using the bicinchoninic acid (BCA) method. The human tissues were provided by the New South Wales Tissue Resource Centre and the Sydney Brain Bank. The regions available for analysis were the hippocampus, frontal cortex (grey matter and white matter), temporal cortex (grey matter and white matter) and cerebellum.

### Western blotting

The brain homogenates were analysed by SDS-PAGE (~15 to 50 μg protein per lane) and western blotting using antibodies to apoD (goat polyclonal sc-34760, Santa Cruz, 1:5,000 for mouse apoD detection; mouse monoclonal, sc-373965 (clone C1), Santa Cruz, 1:1,000, for human apoD detection), apoE (goat polyclonal 178479, Merck Millipore, 1:5,000), NeuN (mouse monoclonal (cloneA60), MAB347, Chemicon 1:1,000), GFAP (rabbit polyclonal, Z0334, DAKO, 1:5,000), MBP (rabbit polyclonal, OABB00147, Aviva Systems Biology, 1:5,000), Iba-1 (rabbit polyclonal, 019–19741, Wako, 1:2000), ZO-1 (rabbit polyclonal, 40–2200, Invitrogen, 1:1000), GAPDH (rabbit polyclonal, OSG00032W, Osenses, 1:10,000) and β-actin (rabbit polyclonal, A5060, Sigma, 1:10,000). Signals were detected using species-specific HRP-conjugated secondary antibodies (all from donkey, Santa Cruz, 1:5,000) and enhanced chemiluminescence and quantified using ImageJ software. Integrated optical density data were normalized to β-actin or GAPDH levels, as appropriate, and expressed as relative values. All quantitative data are presented as mean ± SE (represented by the error bars).

### Sample deglycosylation

Where indicated, selected samples were treated with either peptide N-glycosidase F (PNGase) or neuraminidase (NMDase) to examine the effect of N-glycans and sialylation on apoD relative molecular weight (MW). Tissue homogenates, and/or plasma (pre-diluted with PBS 1:100) were treated with PNGase F (glycerol free, P0704L, NEB) or NMDase (N3786, Sigma) following the manufacturer’s instructions. Sample controls were treated using the same process but without PNGase F or NMDase. All samples were heated for 100°C for 5 min then assessed by 15% SDS-PAGE and western blotting for apoD detection as described above.

## Results and Discussion

**Fig 1:**
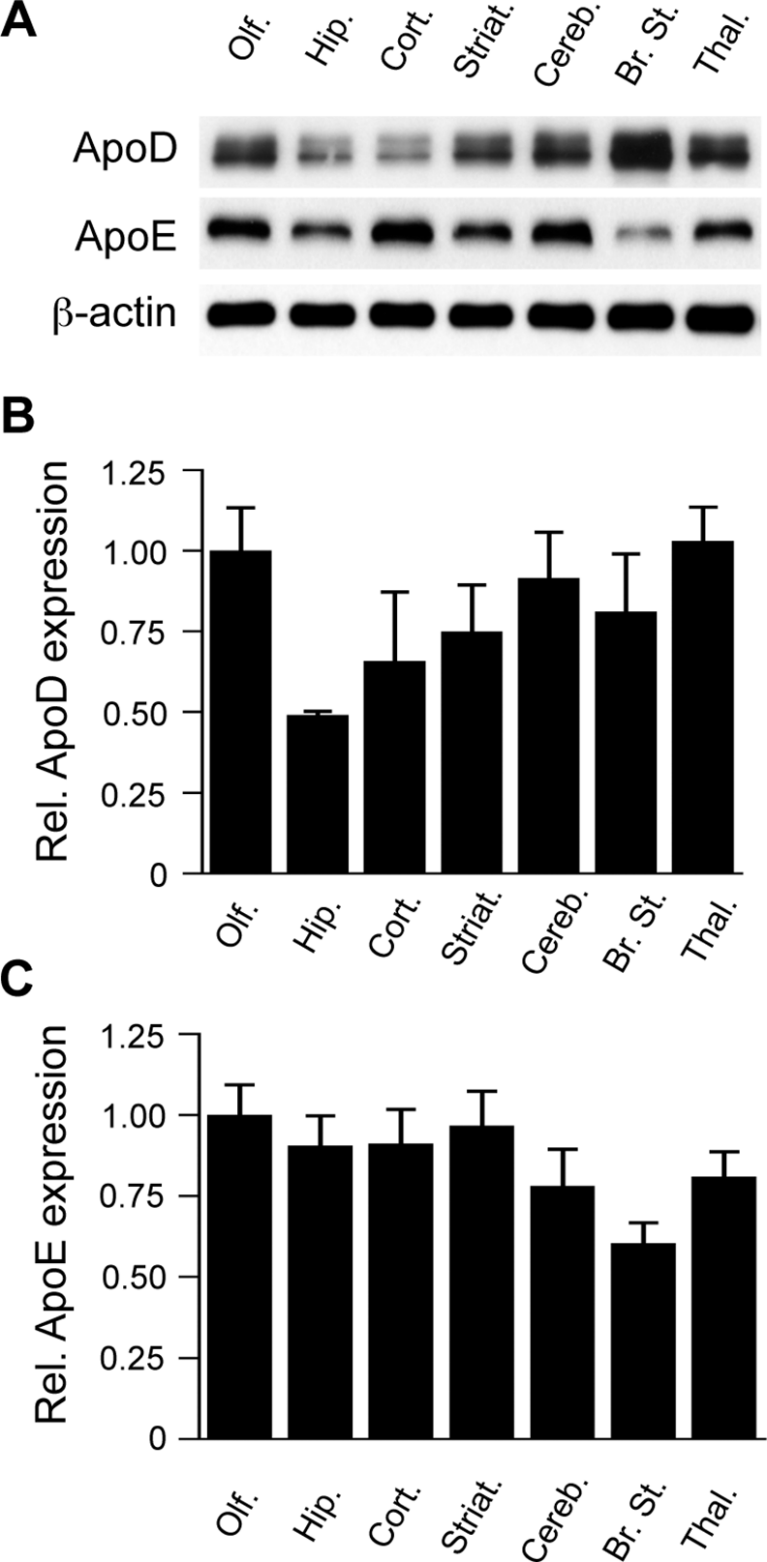
Expression of apoD and apoE in mouse brain regions. The indicated regions of mouse brain were homogenized and assessed for apoD and apoE by western blotting after controlling for equal total protein loading. Levels of β-actin were similar across the brain regions and were therefore used as a housekeeper control protein to normalize apoD and apoE expression. Representative blots are shown from an ~8 month-old male mouse (A). Histograms indicate the relative expression of apoD (B) and apoE (C), corrected for β-actin levels. Data in “B” and “C” are relative optical density measurements where the olfactory bulb is arbitrarily defined as 1.0. The data are derived from 3 different animals (mean values, SE shown by the error bars). Olf., olfactory bulb; Hip., hippocampus; Cort., frontal cortex; Striat., striatum; Cereb, cerebellum; Br. St., brain stem; Thal., thalamus/hypothalamus.

Although apoD is well known to be expressed in the brain, very little has been reported regarding possible differences in apoD protein expression in different brain regions. Using the mouse as a widely studied model organism, we initially examined apoD expression in the following seven major brain regions: olfactory bulb, hippocampus, frontal cortex, striatum, cerebellum, brain stem and thalamus/hypothalamus (the thalamus/hypothalamus regions were combined). Our western blot data indicated variability in the expression of apoD across these brain regions (Fig 1). Of note, the hippocampus and frontal cortex were relatively deficient in apoD compared to most of the other regions (Fig 1A and 1B). For the purpose of comparison, we also examined the expression of apoE in the same brain regions and found a rather different profile. ApoE levels were overall not as variable across the brain regions; with the exception being the somewhat lower apoE level detected in the brain stem (Fig 1A and 1C). Even though apoD and apoE are both regarded as transporters of lipids and other lipophilic molecules [1, 35], their divergent expression patterns in the different brain regions studied here suggests they may have specialised as well as overlapping functions. This is also in line with the divergent expression profiles of these two apolipoproteins in the maturation and ageing of the human brain [25].

**Fig 2:**
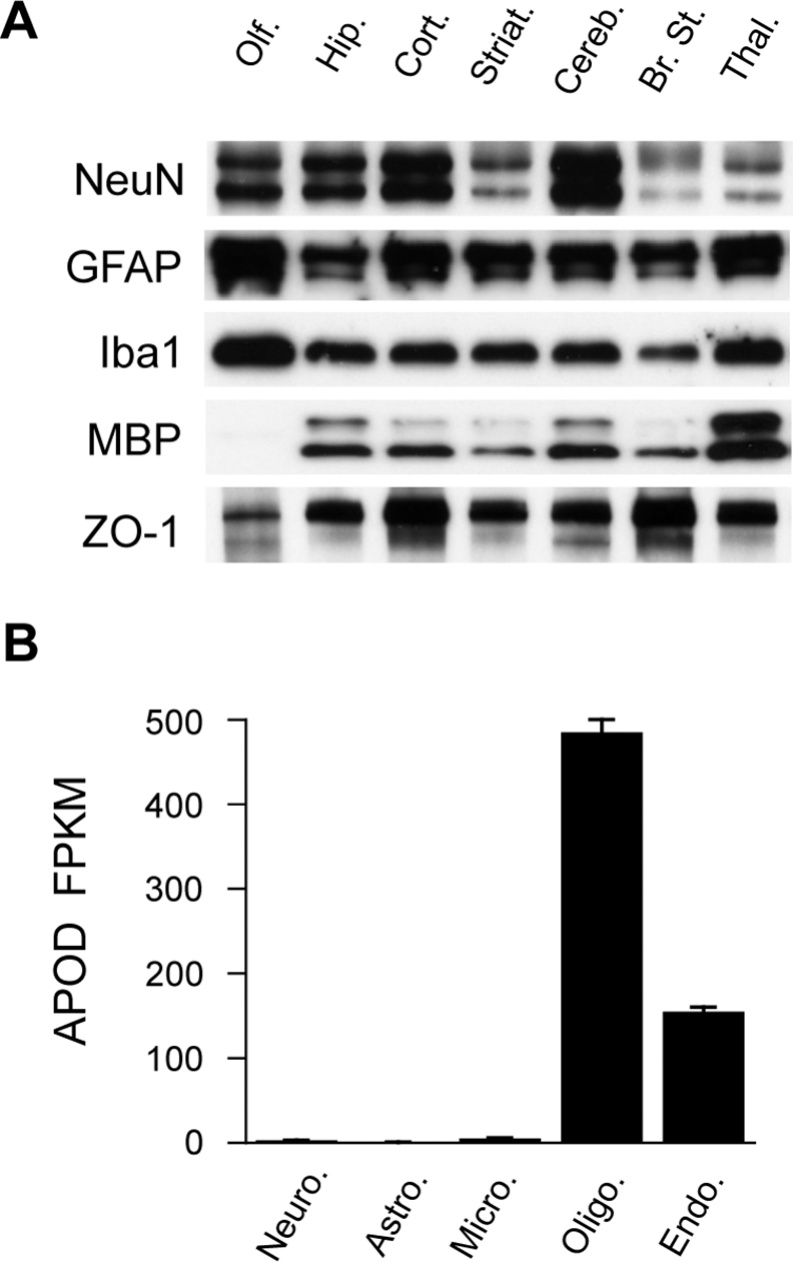
Expression of cell type marker proteins in mouse brain regions. The indicated regions of mouse brain were homogenized and assessed for listed cell specific markers by western blotting after controlling for equal total protein loading. Representative blots are shown from an ~8 month-old male mouse (A). The histogram indicates the predicted relative expression levels of apoD mRNA in various brain cell types (B). Fragments per kilobase of exon per million fragments mapped (FPKM) data are mean values, (SE shown by the error bars) derived from the publically available data based described by Zhang *et al*. (see reference [40]). Olf., olfactory bulb; Hip., hippocampus; Cort., frontal cortex; Striat., striatum; Cereb, cerebellum; Br. St., brain stem; Thal., thalamus/hypothalamus; NeuN, neuronal nuclei; GFAP, glial fibrillary acidic protein; Iba1, ionized calcium-binding adapter molecule 1; MBP, myelin basic protein; ZO-1, zonula occludens protein 1.

Previous studies provide evidence for apoD expression in glia and, under certain circumstances, in neurons [1, 36]. To assess whether the expression of apoD in different brain regions might be associated with enrichment with certain brain cell types, we also probed for a panel of cell-type specific marker proteins. The markers we chose were: neurons, NeuN; astrocytes, GFAP; microglia, Iba1; oligodendrocytes, MBP; and endothelial cells, ZO-1. As expected, the expression of these individual markers varied across the different brain regions (Fig 2A); however, the integrated optical density for the apoD signal was not significantly correlated with any of the individual marker proteins (data not shown). This might suggest that apoD serves different roles in different cell types and that this is in a brain region-specific manner. For example, the higher expression of apoD in the olfactory bulb could be associated with olfaction (e.g. odour delivery to neurons), whereas the high level of apoD expression in the brain stem could have more to do with oligodendrocyte function and myelination. Evidence consistent with a function for apoD in both of these scenarios has been previously reported [37–39].

Related to this line of investigation, a recent transcriptomics study [40], has provided a database of the mouse cerebral cortex transcriptome using a quantitative mapping method that expresses gene expression as fragments per kilobase of exon per million fragments mapped (FPKM). Interrogation of this dataset indicates that at the transcript level *Apod* expression is predicted to be highest in myelinating oligodendrocytes, with the next highest level of expression detected in endothelial cells (Fig 2B). This suggests a role for apoD in myelination, which has also been suggested in other publications [33, 41].

**Fig 3:**
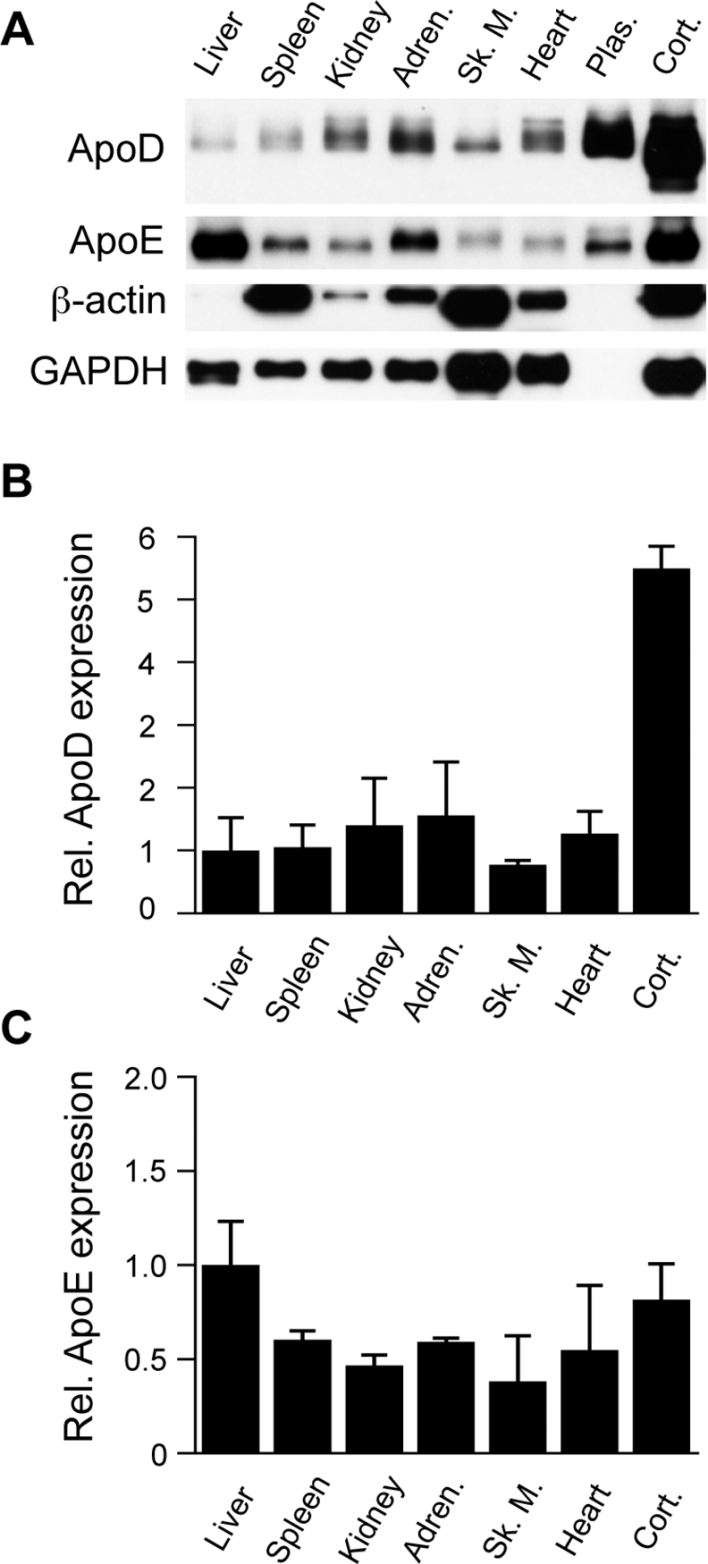
Expression of apoD and apoE in mouse tissues. The indicated tissue samples were homogenized (or collected in the case of plasma) and assessed for apoD and apoE by western blotting after controlling for equal total protein loading. Levels of β-actin and GAPDH were also measured as potential housekeeper proteins. As the GAPDH levels were less variable than β-actin, the former was used to normalize apoD and apoE expression. Representative blots are shown from an ~8 month-old male (A). Histograms indicate the relative expression of apoD (B) and apoE (C), corrected for GAPDH levels. Data in “B” and “C” are relative optical density measurements where the liver is arbitrarily defined as 1.0. The data are derived from 3 different animals (mean values, SE shown by the error bars). Adren., adrenal gland; Sk. M., skeletal muscle; Plas., plasma; Cort., frontal cortex.

In order to compare the level of apoD expression in the brain with other mouse tissues and organs, we compared cortical homogenates with various tissue samples that were collected at the same time the brain dissection was conducted. The organs / tissues we assessed were: liver, spleen, kidney, adrenal gland, skeletal muscle, heart and plasma. With the exception of the plasma samples, these tissues were homogenised using the same protocol we used for the brain regions. Overall, we found that apoD levels were expressed at very low level in all the tissues examined (Fig 3A). In agreement with previous data [2, 12], apoD was expressed at relatively high levels in plasma. When we attempted to quantify the apoD levels in these different tissues using β-actin as a housekeeper protein, the data were highly variable based on the non-uniform expression of β-actin in the samples (Fig 3A). We therefore chose GAPDH as a more reliable housekeeper for these samples. The data indicate that, relative to GAPDH levels, apoD is expressed at the highest level in the brain (Fig 3B). ApoE, on the other hand, was expressed at high levels in both the liver and the brain as well as being detectable at moderate levels in the other tissues examined including plasma (Fig 3C). Once again this indicates that these apolipoproteins are likely to have quite distinct functions that are dependent on the local tissue context.

**Fig 4:**
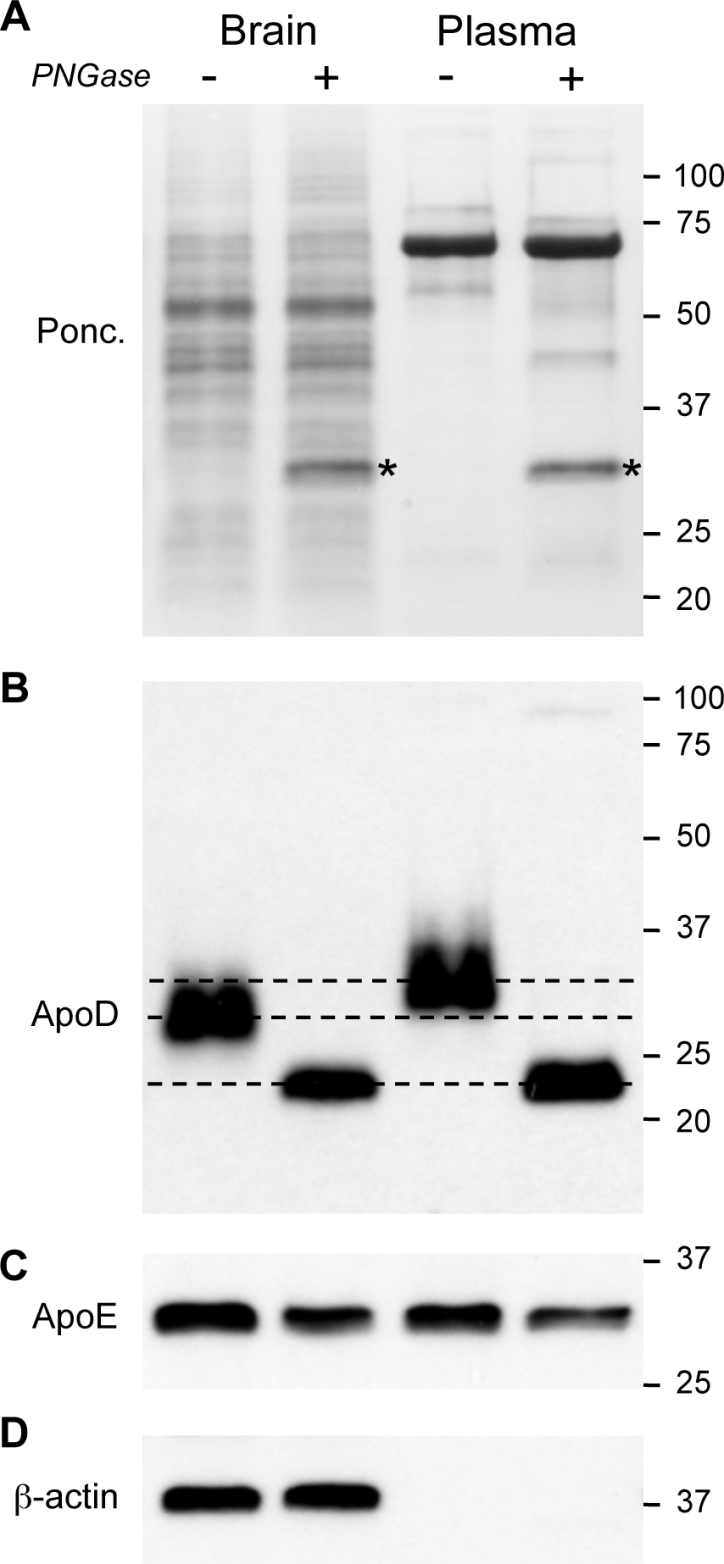
Comparison of brain and plasma apoD molecular weight and impact of N-glycan removal. Mouse brain and plasma samples were run on PAGE under conditions that yielded approximately equal apoD loading. Note that total protein loading was ~50% higher in the plasma samples based on Ponceau staining (A). Both brain and plasma samples with pre-incubated with PNGase (“+”) or buffer control (“-“) to assess the impact of N-glycans on overall MW (B). The broken lines in “B” clarify the migration distances of the different samples and indicates that N-glycans contribute more to MW for the plasma sample. Our comparision apolipoprotein (apoE) was not affected by PNGase, as predicted due to the lack of N-glycans on this protein (C). β-actin levels confirm equal sample loading amounts for the brain samples (D). *, Band due to added PNGase. PNGase, peptide N-glycosidase F.

Our analysis of apoD in the cortex as compared to the various tissues mentioned above indicated an apparent reduced apparent MW of the protein in the brain samples (Fig 3A). This was particularly noticeable in the plasma and cortex samples run beside each other in Fig 3A. To assess whether this difference was due to a truncated form of apoD in the brain or perhaps due to alterations in apoD N-linked glycosylation, we repeated the analysis using PAGE and western blotting conditions that more clearly separated the samples. We also aimed to equalise the loading for apoD level rather than total protein level in this analysis. The total protein loading for this analysis is indicated by the Ponceau Red-stained blots (Fig 4A). The western blot for apoD confirms that there was a striking difference in the apparent MW of apoD in the brain (Fig 4B). We estimate that cortical apoD is ~2 kDa lower in MW than in plasma and peripheral tissues. To assess whether these differences may be due to the extent of N-glycosylation, we treated the samples with PNGase. This treatment resulted in an identical migration position of for both brain and plasma apoD on the blots, thus confirming that the reduced size of brain apoD is indeed due to altered N-glycosylation. No such differences in apparent MW were observed for our comparison protein, apoE, which is an O-glycosylated protein (Fig 4C).

**Fig 5:**
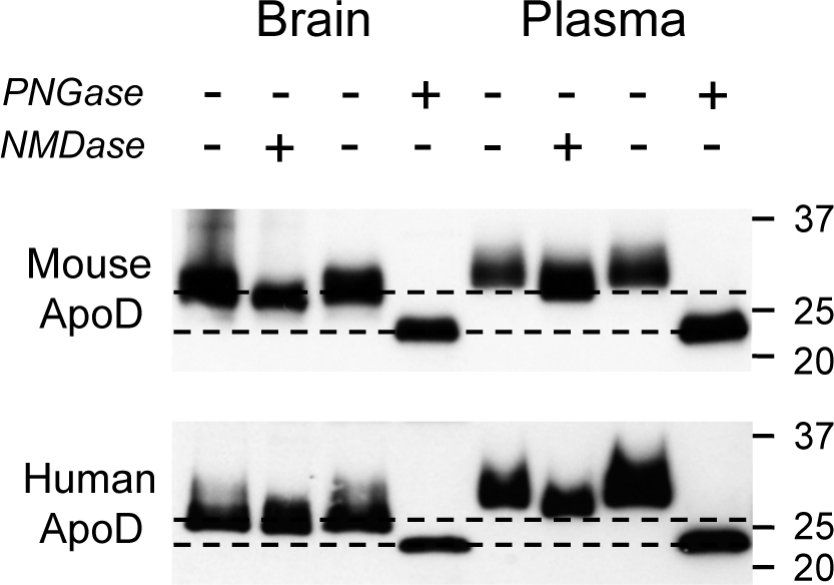
Comparison of mouse and human apoD molecular weight and impact of N-glycan and sialic acid removal. Mouse and human brain and plasma samples were separated by PAGE under conditions that yielded approximately equal apoD loading. Both brain and plasma samples were pre-incubated with either PNGase (“+”) or NMDase (“+”), or the appropriate buffer controls (“-“), to assess the impact of N-glycans and sialic acids, respectively, on overall MW. The broken lines clarify the migration distances of the different samples and indicates that the differences in MW (comparing brain and plasma samples) are not due to sialylation alone (as the MW of plasma apoD is still greater than that of brain apoD after sialic acid removal). In addition, the difference between brain and plasma apoD MW identified in the mouse samples are recapitulated in the human brain and plasma samples. PNGase, peptide N-glycosidase F; NMDase, neuraminidase.

We next assessed the degree to which altered N-glycan sialylation may contribute to the differences in brain apoD glycosylation we detected. Mouse plasma and brain apoD samples were treated with neuraminidase and probed by western blotting. The removal of terminal sialic acids resulted in a “collapsing” or “focusing” of the apoD bands in both the plasma and brain samples; however, the desialylated brain apoD still migrated at a lower MW than the desialylated plasma apoD (Fig 5). PNGase treatment was also used as a positive control for N-glycan removal and, as expected, resulted identical MW products for both plasma and brain apoD. This indicates that the altered apoD N-glycosylation seen in brain apoD is not simply due to the extent of sialylation.

Since mouse and human protein glycosylation may not always be identical (e.g. mouse sialylation involves N-glycolylneuraminic acid whereas human sialylation is due to N-acetylneuraminic acid [42]), we also compared apoD from human plasma and frontal cortex and found the western blot migration pattern was identical to the mouse. Fig 5 also shows that removal of sialic acids from human apoD leads to the predicted drop in MW but the human brain apoD still migrates at a lower MW than the human plasma apoD. Recapitulating the findings from the mouse sample analysis, treatment of human brain apoD and human plasma apoD with PNGase resulted in an identical migration position for the deglycosylated proteins (Fig 5).

Based on data from many previous studies indicating a role for protein N-glycan composition in the regulation of neural transmission and pathways involved in neurodegeneration [43, 44], we speculate that the altered N-glycosylation of apoD seen in the brain, versus other organs, plasma and peripheral tissues, may be of functional significance. Clearly, more detailed fine mapping of the apoD carbohydrate structures and their role(s) in regulating one or more of the postulated functions for apoD would be required to support this conjecture.

**Fig 6:**
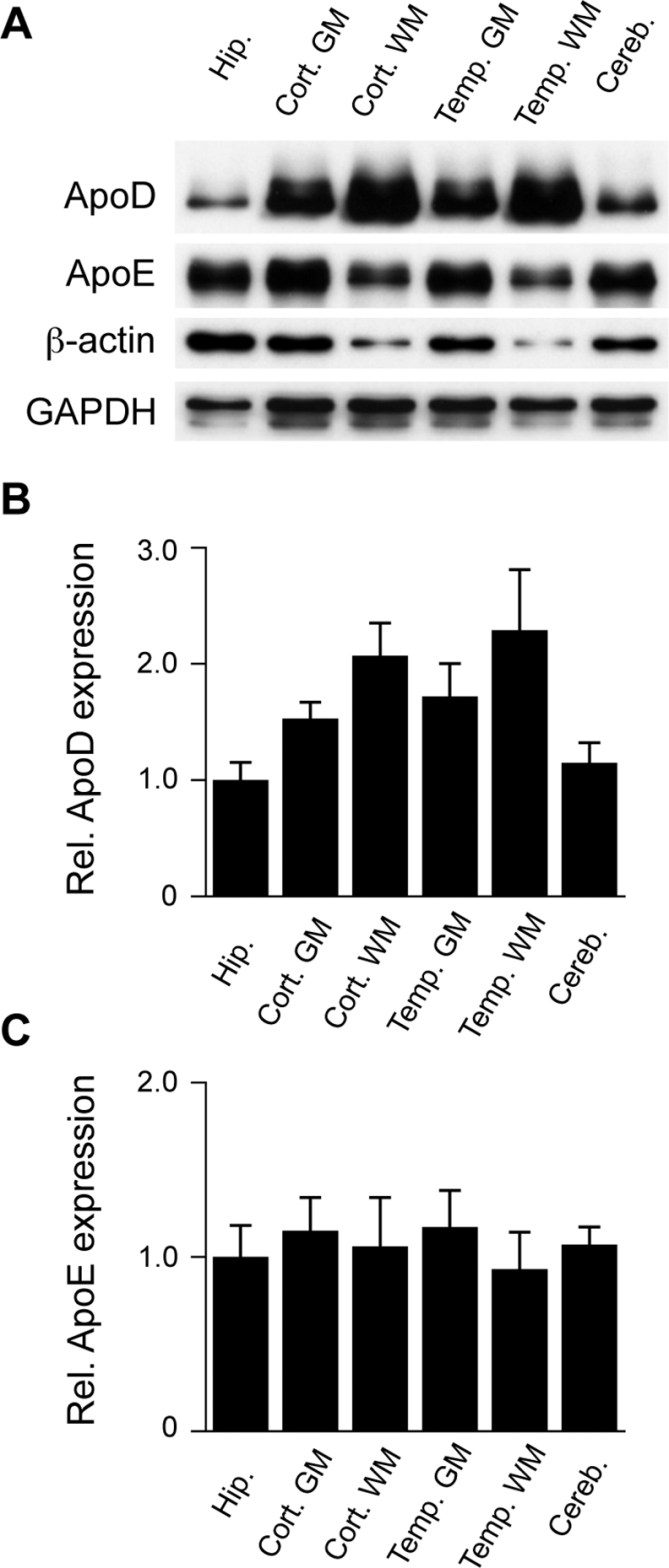
Expression of apoD and apoE in human brain regions. The indicated regions of human brain were homogenized and assessed for apoD and apoE by western blotting after controlling for equal total protein loading. Levels of β-actin were similar across the brain regions and were therefore used as a housekeeper control protein to normalize apoD and apoE expression. Representative blots are shown from a healthy 68 year-old male subject (A). Histograms indicate the relative expression of apoD (B) and apoE (C), corrected for β-actin levels. Data in “B” and “C” are relative optical density measurements where the hippocampus is arbitrarily defined as 1.0. The data are derived from 3 different individuals (mean values, SE shown by the error bars). Hip., hippocampus; Cort., frontal cortex; GM, grey matter; WM, white mater; Temp, temporal cortex; Cereb, cerebellum.

In a final series of experiments we examined the possible variability in apoD expression levels in different regions of human brain samples. We were unable to match identically the same brain regions we used in the mouse brain; nonetheless, a selection of various regions was available to us. In this analysis we have assessed apoD expression in the following brain regions: hippocampus, frontal cortex (grey matter and white matter), temporal cortex (grey matter and white matter) and cerebellum (Fig 6). Similar to the mouse brain region analysis, we found that the hippocampus had the lowest levels of apoD in the human brain regions examined (Fig 6B). We also noted that the apoD expression tended to be higher in the white matter of both the frontal cortex and temporal cortex as compared to the respective grey matter samples (Fig 6B). Also similar to the mouse brain data, the expression of apoE across the human brain regions was not as variable as apoD (Fig 6C). This data therefore confirms the overall observation that apoD expression is variable in different brain regions. Further studies will be required to ascertain exactly what the functional implications of this variable expression might be. As apoD has recently been found to combat lipid oxidative stress in the brain [18], it is possible that regions with high basal apoD may be protected from neurodegeneration whereas regions with low apoD may be more susceptible. In this context it is interesting to note that apoD affords protection in an AD mouse model [19], and the low hippocampal apoD expression we report herein could be one reason that this brain region is highly susceptible in AD. On the other hand, it has also been suggested that the brain stem may be protected from age-related neurodegeneration by virtue of its high basal expression of apoD [45]. Whether the changes in apoD expression that are assocaiated with age and AD are affected equally across all brain areas remains to be established. These research questions, along with the identification of the physiological ligands and functions of apoD in the brain, would appear to warrant further study.

## Conclusions

In conclusion, the present study reveals highly variable expression of apoD in different regions of the mouse brain and human brain. We also show that the apparent MW of apoD is reduced in the brain in both species and that this is due to differences in the apoD N-glycan content. Such differences in apoD regional expression may predispose certain areas of the brain to neurodegenerative disease and/or oxidative stress.

## Abbreviations

AA: arachidonic acid
AD: Alzheimer’s disease
Adren.: adrenal gland
apoD: apolipoprotein D
apoE: Apolipoprotein E
BCA: bicinchoninic acid
Br. St.: brain stem
Cereb: cerebellum
Cort.: frontal cortex
FPKM: fragments per kilobase of exon per million fragments mapped
GAPDH: glyceraldehyde-3-phosphate dehydrogenase
GFAP: glial fibrillary acidic protein
GLaz: glial lazarillo
HDL: high-density lipoprotein
Hip.: hippocampus
Iba1: ionized calcium-binding adapter molecule 1
MBP: myelin basic protein
MW: molecular weight
NeuN: neuronal nuclei
NMDase: neuraminidase
Olf.: olfactory bulb
PBS: phosphate buffered saline
Plas.: plasma
PNGase: N-glycosidase F
Striat.: striatum
Thal.: thalamus/hypothalamus
ZO-1: zonula occludens protein 1
